# Legitimate and Illegitimate Medicine

**Published:** 1868-07-15

**Authors:** W. T. Akins


### LEGITIMATE AND ILLEGITIMATE MEDICINE.

BY W. T. AKINS, M.D.

Prof. Allen:— I have steadily abstained from annoying you with any of my maundering effusions, fearing that some of your readers would deem such asthenic contributions out of place in your valuable columns, and, mayhap, detrimental to medical interests. But I have at last yielded to the solicitations of some friends, and concluded to expend a part of the excess of the *vis nervosa* in inditing you some news and observations, as per heading. I do not propose furnishing your readers with sphygmographic measurements of the professional pulse, or hæmadynamometric calculations as to the medical circulatory forces, nor will I present any standard of ethics born of my ontological views, to which I deem it necessary every man should conform, or suffer the alternative of proscription. But as my article is to suffer no constriction in consequence of old adhesions or recent morphological changes, I will say, *in limine*, "*qui se sent galeux se gale.*" With regret do I announce, as the result of my observations, that the genius, the spirit, and the form of medical ethics, are entirely ignored in the Garden City. The sharp conflict, heated rivalry, and ofttimes base competition that characterize medical as well as other pursuits, has something to do with this lamentable state of affairs. Another and perhaps stronger cause, is the presence here of so many ignorant pretenders —

Prof. Rea's class of "detested hurters," with whom high-minded, educated medical men will never fraternize. You may have magnanimity of heart, and catholicity of spirit, and yet you will find it hard beyond the possibility of consummation to counsel with or recognize these lilliputian hegations. Treat them with conversational regard, and they will insult you by sycophancy and servility, mutilations of language, and ignorance of philosophy. They will use synonymously pathology and physiology, confound retention and suppression as words expressive of similar conditions, and cap the climax by holding proctelitis and phrenitis to be one and the same disease. Shades of Apollo! Royal son of Delos! Just think of one of these molecules of a professional micros reading the Sybilline leaves of destiny to some fated mortal, without as much as consulting the degrees of Parcæ. Here are Bassett and Bigelow, *et id omne genus*, each of them a veritable Autolycus, stealing the gifts of legitimate medicine, and prostituting all to the base and shameful purposes for which they live. It is sadly to be feared that the Danaides will accomplish their task in the regions of Tartarus, ere we succeed in cleaning this Augean stable of ours, unless a wiser legislation and a higher regard for ethics renders it impossible for these impostors to get license or business. Perhaps the most convincing testimony as to the profound ignorance of some who claim to be regulars, is to be found in their opinions and remarks. Dr. Clark was lucky enough to get hold of a prescription by one of these benighted fellows, and gave it *in toto*, to show us a specimen of our rivals. Less fortunate than the Dr., I can only give you the remarks and opinions as made and offered verbally. One of these fellows, reputed to be the surgeon of a semi-quack establishment, expressed it as his opinion that ossification of the "ductus arteriosus" contributed largely to the production of hypertrophy of the heart. That there was a great difference between scarlatina and scarlet fever. That the antero-posterior diameter of a normally developed fœtal head at birth is nine inches. During the prevalence of small pox last winter, one of these fellows, formerly a plasterer in

St. Paul, visited a woman, and notwithstanding the patient was suffering from great pain in the back, or spine-ache, distressing nausea and vomiting, severe cephalalgia, hot skin, and accelerated pulse, he pronounced it a bad cold; visited her next day, and although all the symptoms were more aggravated, confirmed his diagnosis, and left. I was called in a few hours, and found the patient laboring under a severe attack of variola confluens, eruption thickly studding corpus and temporal region. Two other notables of the same class visited — shades of tocology! — a woman in confinement, and after concluding to rupture the membranes, laid open the scalp from the coronal to the lambdoidal suture, down to the skull. It is burdensome to offer more, and all must be disgusted ere this. One, called a surgeon, without information enough to perform the duties of an oncotomist; the other practicing medicine without being able to diagnose a disease with every diacrittic symptom needful to a tyro; and, finally, these tocologists, unable to distinguish between the membranes and the fœtal scalp. Turning about a little, and I recognize still another, who, though proclaimed a Coryphœus in the healing art, I can not but look upon as very much less than an "apostle," if I am to understand by the word apostle, one who teaches and practices the doctrines of the great I AM. It may not be needful, in order to the honorable and skillful practice of physic, that every practitioner should make stated pilgrimages to the Castalian fount, where poets drank and muses quaffed, damp his brow with dews of Helicon, or rest him awhile in some parterre on the sacred mount of Parnassus; but ere he proclaims himself the only medical reformer of his time and age, is it not well that he look a little after all the needed accomplishments calculated to aid him in his high and hard battlings for his favorite dream; that he manifest consummate mastery of facts, analysis, and logic, in his discourses on medical philosophy, and that he exemplify in his practice the unfailing worth of the truisms and axioms promulgated in his teachings. And, furthermore, would not a more rigid regard for ethics, and the feelings and ability of others, contribute to his honor, and the number of his proselytes? By some he is acknowledged as the great medical Cyclops, and he may be; but his foibles are as apparent as was the heel of the armor-encased Achilles. You may wonder why one unknown to fame presumes thus to touch the little fishes that sport in the shoals, and at the same time lay hands on the great leviathan of the unfathomed deep. I can only say that as a member of our profession, and wishing to see its ranks depleted of impostors, and recruited by able, scholarly men, I felt it my right and my duty to speak independently and fearlessly. The rich mines of English, German, and French medical literature are open to all of us who have the mental hardihood to win our way to the feast; and for any man to arrogate to himself the possession of all knowledge found in the wide domain of medicine and surgery, is to discover to those about him his utter unfitness to teach or practice. In some future number I will present my views, if acceptable, as to the best and most expedient method of securing ourselves and the people against the ignorance of the puerile, and the impudence of impostors. I have written in no spirit of enmity or hate, but from candor, and from deep convictions. We must devise some method by which we may free our ranks of the many disgracefully ignorant who practice under the title of "regular," and the community of the host of quacksalvers, who prey like insatiate vultures upon the vitals of community — and certainly the people would soon be annihilated but for the fiat, *Crescite et mutiplicamini*, which enables them, Prometheus like, to reproduce themselves.

